# Type 4 Dual Left Anterior Descending Artery: A Very Rare Coronary Anomaly Circulation

**DOI:** 10.1155/2015/580543

**Published:** 2015-04-08

**Authors:** Marcos Danillo Peixoto Oliveira, Pedro H. M. Craveiro de Melo, Antonio Esteves Filho, Luiz J. Kajita, Expedito E. Ribeiro, Pedro Alves Lemos

**Affiliations:** Department of Interventional Cardiology, Heart Institute of the University of São Paulo, Avenida Dr. Enéas de Carvalho Aguiar 44, 05403-900 São Paulo, SP, Brazil

## Abstract

Coronary artery anomalies are congenital changes in their origin, course, and/or structure. They are the second most frequent cause of sudden death in young athletes. Dual LAD artery is a rare coronary anomaly. We present the case of a 44-year-old man with recent onset exertional angina and documented ischemia whose coronary angiogram and computed tomography (CT) showed type 4 dual LAD artery, the rarest and most interesting variant.

## 1. Introduction

Coronary artery anomalies (CAA) are a diverse group of congenital disorders, and the pathophysiological mechanisms and manifestations are highly variable. Its incidence ranges from 0.03% to 0.2% of the patients undergoing routine catheterization [1]. Dual left anterior descending (LAD) artery circulation is an uncommon coronary anomaly. In type 4, the rarest, one artery arises from the right sinus of Valsalva or the right coronary artery (RCA) [2].

## 2. Case Report

**Figure 1:**
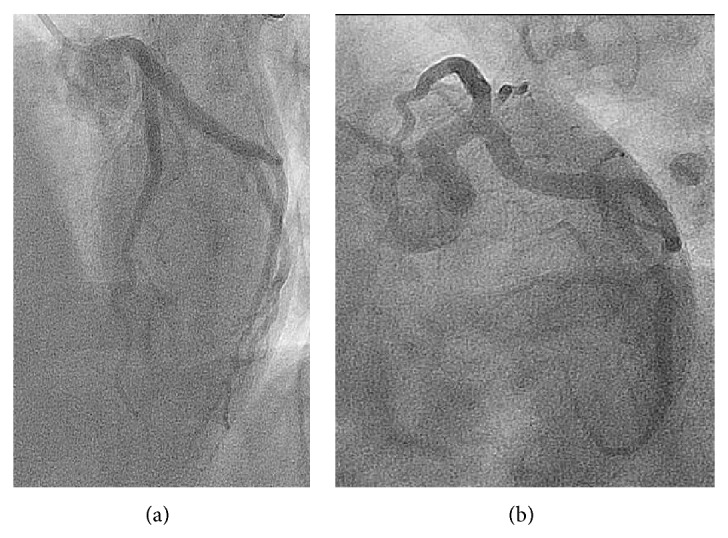
LCA, with the “short” LAD artery coursing through the proximal part of the AIVS. (a) LAO cranial view. (b) LAO caudal “spider” view. LCA, left coronary artery; LAD, left anterior descending artery; AIVS, anterior interventricular sulcus; LAO, left anterior oblique.

**Figure 2:**
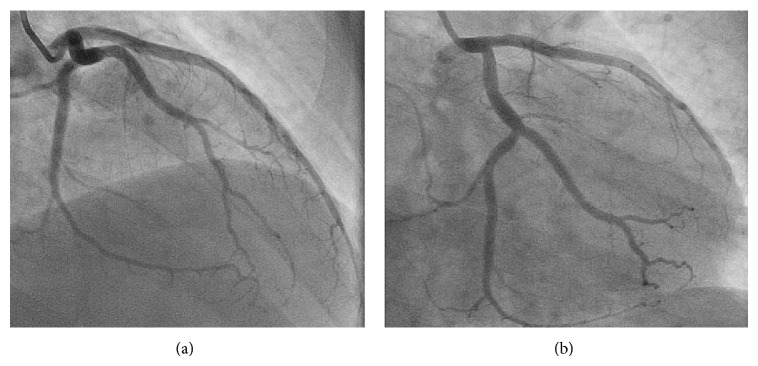
LCA, with the “short” LAD artery coursing through the proximal part of the AIVS. (a) RAO caudal view. (b) PA caudal view. LCA, left coronary artery; LAD, left anterior descending artery; AIVS, anterior interventricular sulcus; RAO, right anterior oblique; PA, posterior-anterior.

**Figure 3:**
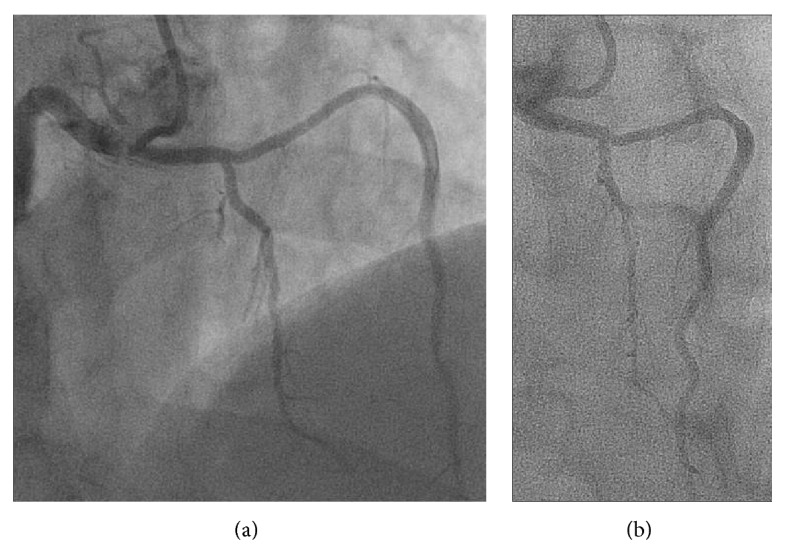
The “long” LAD artery, from the right sinus of Valsalva, close to the RCA ostium (a), until the distal part of the AIVS, after the emergence of a large septal branch (b). LAD, left anterior descending artery; RCA, right coronary artery; AIVS, anterior interventricular sulcus.

**Figure 4:**
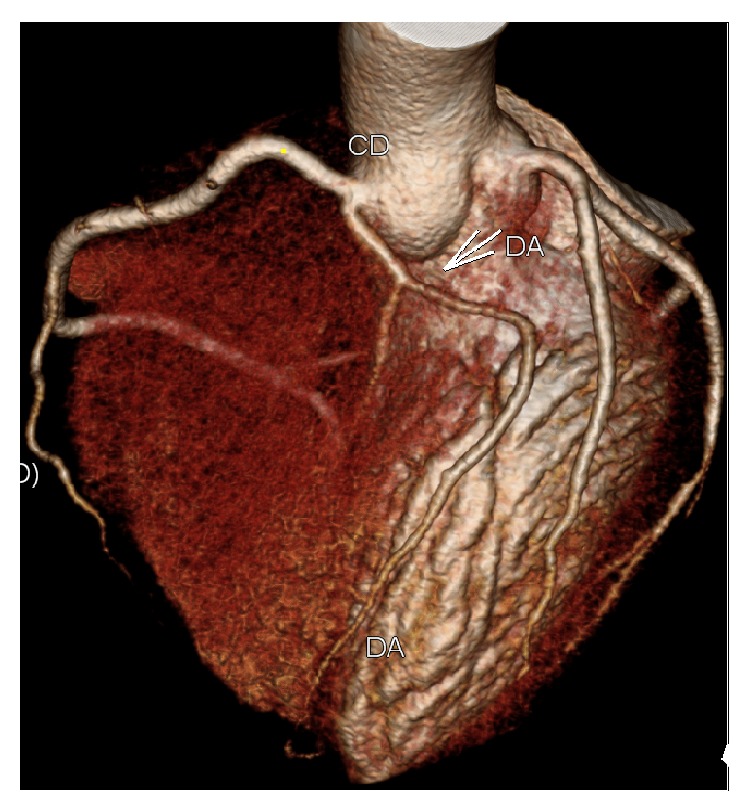
CT angiography confirming the anomalous (interarterial) course of the long LAD artery (white arrow) from the right sinus of Valsalva (close to the RCA ostium), coursing along the distal part of the AIVS (the pulmonary trunk was removed). CT, computed tomography; LAD, left anterior descending artery; RCA, right coronary artery; AIVS, anterior interventricular sulcus.

A 44-year-old man, active, Caucasian, presented with recent onset exertional angina. The resting electrocardiogram (ECG) was normal and a stress echocardiogram revealed normal left ventricular function and significant anterior ischemia. He underwent an angiogram that revealed the rare coronary anomaly, with two LAD arteries. One normally originated from the left coronary artery (LCA), coursing through the proximal part of the anterior interventricular sulcus (AIVS) and ending well before the apex (“short LAD artery”), but after the emergence of various small septal branches (Figures 1 and 2). Another LAD artery originated from the right sinus of Valsalva (close to the RCA ostium), coursing along the distal part of the AIVS, after the emergence of a large septal branch (“long LAD artery,” Figure 3). No significant stenotic lesion was noted in any of the coronary arteries. This coronary pattern is consistent with type 4 dual LAD artery [2]. In order to better define the “long LAD artery” course and its relationship with adjacent structures, a computed tomography (CT) angiography was performed. It confirmed the anomalous (also called malignant) course of the long LAD artery between the aorta and the pulmonary trunk (Figure 4). The patient was advised about the implications of this diagnosis and was discouraged to the practice of intense or competitive physical activities. He refused the proposed coronary bypass surgery. Ten months after the coronary angiogram, he remains stable and adherent to the medical recommendations, with relief of symptoms under beta-blocker therapy, without documentation of ischaemia or arrhythmias on a new control exercise test.

## 3. Discussion

CAA are congenital changes in their origin, course, and/or structure. Several controversies remain in terms of its incidence, classification, screening, heredity, and treatment. Despite being mostly asymptomatic, clinical presentation in adults may result from myocardial ischemia, manifesting as angina, syncope, arrhythmias, and even sudden death. In young athletes, apparently healthy, they are the second most frequent cause of sudden death [3].

Dual LAD artery is a rare coronary anomaly. One artery runs along the proximal part of the AIVS but stops well before the apex (“short LAD” artery) while the second one joins the AIVS distally and reaches the apex after originating elsewhere (“long LAD” artery) [2]. Spindola Franco et al. [4] described four types of dual LAD artery. In the first three types, the “short” and “long” LAD arteries arise from the LCA. However, the rarest and most interesting variant is type 4 in which the “short LAD” artery arises from the LCA while the “long LAD” artery arises from the RCA or the right sinus of Valsalva.

This rare anomaly can potentially present with unusual clinical features and also lead to confusion on the angiogram where a “short LAD” artery may be misdiagnosed as a total occlusion [2].

In our case, no lesions were noted in any of the coronary arteries, so the symptoms of angina and the documented ischemia might be a consequence of long LAD extrinsic compression into its interarterial course, between the aorta and the pulmonary arteries, which during exercise dilate and compress the coronary [3].

The guidelines of the American College of Cardiology/American Heart Association for the Management of Adults with Congenital Heart Disease [5] give an indication class I, level of evidence B, for CT angiography and cardiac magnetic resonance imaging (MRI) in the diagnosis of CAA. Considering that the CT angiography is more widely available and allows to properly define its origin, course, path relationships with other anatomical structures, and special advantage over conventional coronary angiography, it seems to be the method of choice in most cases where it is suspected to be CAA [3]. Bypass coronary surgery is indicated (indication class I, level of evidence B) in cases like ours, with the interarterial course of the coronary artery [5].

## 4. Conclusion

This case report reinforces the importance of CAA as a hypothesis in the differential diagnosis of angina and ischemia. Type 4 LAD artery, due to its possible interarterial course, must be early diagnosed and appropriately managed in order to prevent sudden cardiac deaths in young and healthy patients.
